# Peach PpSnRK1 Participates in Sucrose-Mediated Root Growth Through Auxin Signaling

**DOI:** 10.3389/fpls.2020.00409

**Published:** 2020-04-24

**Authors:** Shuhui Zhang, Futian Peng, Yuansong Xiao, Wenru Wang, Xuelian Wu

**Affiliations:** State Key Laboratory of Crop Biology, College of Horticulture Science and Engineering, Shandong Agricultural University, Tai’an, China

**Keywords:** peach, sucrose, lateral roots, SnRK1, IAA

## Abstract

Sugar signals play a key role in root growth and development. SnRK1, as one of the energy centers, can respond to energy changes in plants and affect the growth and development of plants. However, studies on sugar signals and SnRK1 regulating root growth in fruit trees have not been reported. In this study, we found that 5% exogenous sucrose could increase the total volume and total surface area of the peach root system, enhance the number and growth of lateral roots, and promote the activity of SnRK1. When exogenous trehalose was applied, the growth of roots was poor. Sucrose treatment reversed the inhibitory effects of trehalose on SnRK1 enzyme activity and root growth. We also found that the lateral root number of *PpSnRK1a*-overexpressing plants (4-1, 4-2, and 4-3) increased significantly. Therefore, we believe that peach SnRK1 is involved in sucrose-mediated root growth and development. To further clarify this mechanism, we used qRT-PCR analysis to show that exogenous sucrose could promote the expression of auxin-related genes in roots, thereby leading to the accumulation of auxin in the root system. In addition, the genes related to auxin synthesis and auxin transport in the root systems of *PpSnRK1a*-overexpressing lines were also significantly up-regulated. Using peach PpSnRK1a as the bait, we obtained two positive clones, PpIAA12 and PpPIN-LIKES6, which play key roles in auxin signaling. The interactions between peach PpSnRK1a and PpIAA12/PpPIN-LIKES6 were verified by yeast two-hybrid assays and bimolecular fluorescence complementation experiments, and the complexes were localized in the nucleus. After exogenous trehalose treatment, the expression of these two genes in peach root system was inhibited, whereas sucrose had a significant stimulatory effect and could alleviate the inhibition of these two genes by trehalose, which was consistent with the trend of sucrose’s regulation of SnRK1 activity. In conclusion, peach SnRK1 can respond to sucrose and regulate root growth through the auxin signal pathway. This experiment increases our understanding of the function of fruit tree SnRK1 and provides a new insight to further study sugar hormone crosstalk in the future.

## Introduction

Sucrose, as one of the main forms of sugar in plants, plays important roles in growth and development and in stress responses (Lastdrager et al., 2014). Previous studies have reported that the addition of sucrose (50 mmol⋅L^–1^) into the culture medium could reverse the inhibitory effect of micro-RNA156 on flowering (Yu et al., 2013). When sucrose is added into the medium, it can promote plant rooting and germination. In *MED12* (CENTER) and *MED13* (GRAND) *Arabidopsis thaliana* mutants, the root morphology was reversed by exogenous sucrose treatment (Raya-González et al., 2017). Schneider et al. (2019) found that sugar plays a key role in bud outgrowth and is linked to hormones that regulate bud growth. When the content of auxin in plants is changed without changing the content of sucrose, the growth of buds will not change, whereas when the content of sucrose is changed without changing the content of hormones, exogenous sucrose can quickly induce the growth of buds, indicating that sucrose is the first signal to trigger bud outgrowth (Barbier et al., 2015). Research by Christine Beveridge’s lab has found that sugar signals can break bud dorms and regulate apical dominance, which means that the top bud can rob sucrose, depriving the lateral bud of sucrose and inhibiting the growth of the lateral bud, retaining apical dominance. The lab also found a balance between the growth of branches aboveground and roots belowground (Beveridge et al., 2000; Mason et al., 2014). How sucrose regulates root growth remains unknown. In particular for Rosaceae fruit trees, root growth is particularly important. The effect and specific mechanism of sucrose on fruit trees have not been studied.

As an energy sensor, the SnRK1 protein kinase (sucrose non-fermentation-1-protein kinase-1) can sense intracellular sugar levels. It plays important roles in plant growth, metabolism, stress responses, and other processes (Smeekens et al., 2010; Yu et al., 2015; Li and Sheen, 2016). Wurzinger et al. (2018) reported that SnRK1 regulates sucrose production, starch synthesis, and enzyme activity, thereby regulating the carbohydrate metabolism of plants and affecting their growth and development. SnRK1 protein kinase phosphorylates enzymes involved in plant metabolism, such as nitrate reductase (NR), 3-phosphoglyceraldehyde dehydrogenase (np-Ga3PDHase), sucrose phosphate synthase (SPS), and trehalose phosphate synthase (TPS), thereby regulating the energy balance (Piattoni et al., 2011; Nukarinen et al., 2016). Fujita et al. (2006) demonstrated that SnRK1 can inhibit the expression of more than 300 genes during the synthesis of amino acids, sucrose, lipids, and other substances. At the same time, it can promote the expression of nearly 300 genes involved in the catabolism of proteins, cellulose, and polysaccharides. Plants can sense energy signals and regulate complex metabolic processes through SnRK1, so as to make up for deficiencies in energy and to maintain normal activities. Previous studies have found that under conditions of glucose starvation, SnRK1 can induce the expression of α-amylase through MYBS1 to regulate seed germination and seedling growth (Lu et al., 2007). *SnRK1* overexpression in *A. thaliana* can delay flowering time and aging and can cause fruit and cotyledon defects (Tsai and Gazzarrini, 2012). When *SnRK1* was expressed in the antisense, plants showed sensitivity to salt, and taproot growth was inhibited (Lovas et al., 2003). Other studies have also found that SnRK1 associates with hormones, such as ABA, ethylene, and other signaling molecules, to regulate plant metabolism, to regulate growth and development, and to respond to stress (Coello et al., 2012; Kim et al., 2017).

We previously reported that *PpSnRK1a*-overexpressing tomato plants showed an increase in the resistance to stress and a delay in aging (Luo et al., 2017). Intriguingly, transgenic tomato plant lateral root growth was better than that of the wild-type (WT) (data not published). These findings prompted us to investigate whether peach SnRK1, as an energy sensor, could respond to sugar and regulate the growth and development of plant lateral roots. Analysis of the gene expression profiles of *MhSnRK1*-overexpressing tomato lines revealed that *MhSnRK1* overexpression could increase the expression of genes involved in auxin synthesis and transport. Auxin plays important roles in root growth and development. In addition, it regulates taproot growth; promotes the formation of lateral roots, adventitious roots, and root hairs; and induces vascular differentiation. Therefore, sucrose-mediated root growth and development likely involve important interactions between SnRK1 and auxin signaling. Here, we examined the role of sucrose on root growth and development, and we investigated the involvement of SnRK1 and auxin signaling in these processes. Our results provide a foundation for further studies on the cultivation of peach trees.

## Materials and Methods

### Plant Materials and Growth Conditions

This experiment used peach (*Prunus persica*) seedlings, whose seeds were stratified in sand in December 2017. We selected seeds with full bud cracks in April 2018 and planted them in a 50-well seedling plate. After 1 month, peach seedlings with consistent growth were transplanted into a pot (32 cm × 25 cm) containing garden soil, matrix, and vermiculite at a ratio of 2:1:1 (v/v/v). After 30 days, plants with consistent growth were selected and treated. First, we applied sucrose solutions at different concentrations (0, 1, 3, 5, and 7%), and the volume of each solution was 1 L. Three replicates were set for each treatment, with six seedlings. After 7 days of treatment, we measured the activity of SnRK1 enzyme and soluble sugar content in the root system. Water and 5% mannitol were used as controls. In a second experiment, three replicates were set, with 20 plants for each treatment. On day 13, the auxin content in plant roots was determined by high-performance liquid chromatography (HPLC), and root configuration parameters were determined by the WinRHIZO root analysis system. The entire experiment was repeated in 2019.

In this experiment, exogenous trehalose was used to influence the regulatory ability of sucrose on SnRK1. The experiment was divided into short-term treatment (the volume of treatment solution was 250 ml, and the treatment time was 4 h) and long-term treatment (the volume of treatment solution was 50 ml, each solution was treated once every 5 days, five times; and the root phenotype and enzyme activity were measured by sampling on the 30th day), and each treatment was divided into four groups, with each group comprising 10 seedlings. The first group of seedlings was treated with water. The second group of seedlings was treated with 5% sucrose. The third group of seedlings was treated with 50 mmol⋅L^–1^ of trehalose, and the last group was 5% sucrose + 50 mmol⋅L^–1^ of trehalose [1:1 (v/v)]. The experiment was repeated three times independently.

We previously obtained *PpSnRK1*α-overexpressing transgenic lines 4-1, 4-2, and 4-3. Seeds of transgenic and WT (*A. thaliana* “Columbia”) lines were soaked in distilled water for 1 h and disinfected on an ultraclean working table. The seeds were treated with 70% alcohol for 30 s, washed with sterile water four times, treated with 3% sodium hypochlorite for 15 min, shaken three times during soaking, and washed with sterile water five to six times. The seeds were spread on Murashige and Skoog (MS) solid medium, subjected to cold treatment at 4°C for 24 h, and then grown in an artificial incubator with a light cycle of 16 h/8 h, a temperature of 23°C, and a relative humidity of (70 ± 5%). On day 30, root growth was assessed, and gene expression was examined.

### Quantitative Polymerase Chain Reaction

Total RNA was extracted from *A. thaliana* roots using the RNA Plant Plus Reagent Kit (TIANGEN, China) and reverse transcribed using the PrimeScript First-Strand cDNA Synthesis Kit (Takara, Dalian, China). Quantitative polymerase chain reactions (qPCRs) were carried out in the ABI7500 System using SYBR Premix Ex Taq (Takara). AtActin-F/R and PpActin-F/R primers were used to evaluate the expression of *A. thaliana* and *P. persica actin* genes, which served as the internal reference genes (Table 1). The calculation method for qRT-PCR was 2^–ΔΔCT^. At least three replicates for each sample were used for qPCR. The primers used for qPCR are listed in Table 1.

**TABLE 1:** Primers used in this study.

Gene	Prime name	Prime sequence (5′–3′)
*PPIAA12*(ppa009545m)	P-IAA12-S	GTGAGCGTGGTAGAATCTTGATG
	P-IAA12-A	CATCCCACAACTTGACTTACAGC
*PIN-LIKES6*(ppa006242m)	P-PIN6-S	TGGAAAGGTTTTTACAGGTGGTG
	P-PIN6-A	CTGTAAAAACCTTTGCTATGGGC
*PPIN1*(Ppa002944m)	P-PIN1-S	TAACAATACGACAGCGCATTACC
	P-PIN1-A	TGAAGATCCTTACCACCATCCTC
*PPIN2*(Ppa024134m)	P-PIN2-S	TTCGAATCTCACGGGAGTGG
	P-PIN2-A	GAATCCACCTTGGAAACTGTTTG
*PPIN3*(Ppa002528m)	P-PIN3-S	ATCTAACCTTACAGGCGCAGAGA
	P-PIN3-A	GAGTCTCTTCGAAATTTGACGGT
*PPYUC2*(Ppa022204m)	P-YUC2-S	GACCCAGCAGTGTTCGATCA
	P-YUC2-A	CTGCCTCCTCCAATTCTGGCT
*PPYUC6*(ppa005244m)	P-YUC6-S	TCCTCCTCATCACCATCACA
	P-YUC6-A	CCACAAGAGGCTATGCAATT
*AtPIN1*(AT1G73590)	AtPIN1-F	CGGTGGGAACAACATAAGCA
	AtPIN1 -R	CACACTTGTTGGTGGCATCAC
*AtPIN2*(AT5G57090)	AtPIN2 -F	CCGTGGGGCTAAGCTTCTCATCT
	AtPIN2 -R	AGCTTTCCGTCGTCTCCTATCTCC
*AtPIN3*(AT1G70940)	AtPIN3 -F	CGGAGCACCTGACAACGAT
	AtPIN3 -R	CGGATCTCTTTAGCACCTTGGT
*AtTAA1*(AT1G70560)	AtTAA1 -F	GATGAAGAATCGGTGGGAGAAGC
	AtTAA1 -R	CGTCCCTAGCCACGCAAACGCAGG
*AtYUC2*(AT4G13260)	AtYUC2 -F	CAAGGTGTATCCGGAGTTGA
	AtYUC2 -R	AATGGCTGCACCAAGCAATC
*AtYUC6*(AT5G25620)	AtYUC6 -F	TATACGCGGTCGGATTCACA
	AtYUC6-R	CCACCACAATCACTCTCACT
*PPActin*	P-Actin-F	GTTATTCTTCATCGGCGTCTTCG
	P-Actin-R	CTTCACCATTCCAGTTCCATTGTC
*AtActin*	AtActin-F	CGCTCTTTCTTTCCAAGCTC
	AtActin-R	AACAGCCCTGGGAGCATC

### SnRK1 Activity Assays

Approximately 1.0 g of fresh roots was weighed and minced with a high-throughput tissue grinder. Subsequently, 1 ml of pre-cooled extraction buffer was added, and the extraction was performed as described previously (Yu et al., 2018). The AMARA polypeptide served as the substrate (Zhang et al., 2009; Debast et al., 2011), and SnRK1 activity was measured with the Universal Kinase Activity Kit (R&D Systems, Minneapolis, MN, United States).

### Yeast Two-Hybrid Screening and Assays

The full-length *PpSnRK1*α cDNA was cloned into the pGBKT7 vector. Yeast two-hybrid screening of the peach cDNA library was performed according to the manufacturer’s instructions (Clontech, Dalian, China) and then the coating on selection SD medium lacking adenine, histidine, leucine, and tryptophan (SD-A-H-L-T).

For the yeast two-hybrid experiments, pGBKT7 and pGADT7 plasmids were used. *PpSnRK1*α was amplified and then cloned into the pGBKT7 vector, whereas full-length *IAA12* and *PIN-LIKES6* cDNAs were cloned into the pGADT7 vector. Transformed colonies grown on synthetic dropout medium lacking leucine and tryptophan (SD-Leu-Trp) were selected. After 3 days, the colonies were coated with quadruple selection SD medium lacking Trp, Leu, His, and Ade (SD/-T/-L/-H/-A). To further confirm the positive interactions, X-alpha-gal was used to measure beta-galactose activity. The cloning primers for *PpSnRK1*α were *PpSnRK1*α-F (5′-GCTCTAGAATGGATGGATCGGTTG-3′) and *PpSnRK1*α-R (5′-GCGTCGACTTAAAGGACCCG-3′). The cloning primers for *IAA12* were *IAA12*-S (5′-TCCCCCGGGGGAATGTCAAAGGTGGAGGTGGTG-3′) and *IAA12*-A (5′-CGAGCTCGGATAGGTTTGTTTCTTCGTCTC TCAC-3′). The cloning primers for *PIN-LIKES6* were *PIN6*-S (5′-GGAATTCCATGGAAAGGTTTTTACAGGTGGTG-3′) and *PIN6*-A (5′-CGGGATCCCGAAACAGTATGTTGAGGTACAAT ACAATC-3′).

### Bimolecular Fluorescence Complementation Assay

*PpSnRK1*α was cloned into the pSPYCE vector, whereas *IAA12* and *PIN-LIKES6* were cloned into the pSPYNE vector. Positive clones were obtained and transformed into the agrobacterium LBA44404. Protein interactions in tobacco leaves were observed by agrobacterium instantaneous infection, and the fluorescence intensity was monitored under a Zeiss LSM880 ultra-high-resolution laser confocal microscope.

### Statistical Analysis

All results were shown as mean values of three independent experiments. Microsoft Excel 2007 Software was used for data analysis. All samples were analyzed in triplicate and represented as the mean ± standard deviation unless specifically labeled. The comparison of means was carried out by the Duncan multiple range test using SPSS 20.0 software. Significance was defined at *P* < 0.05.

## Results

### Effects of Sucrose Concentration on Soluble Sugar Content and SnRK1 Enzyme Activity in Peach Roots

**FIGURE 1:**
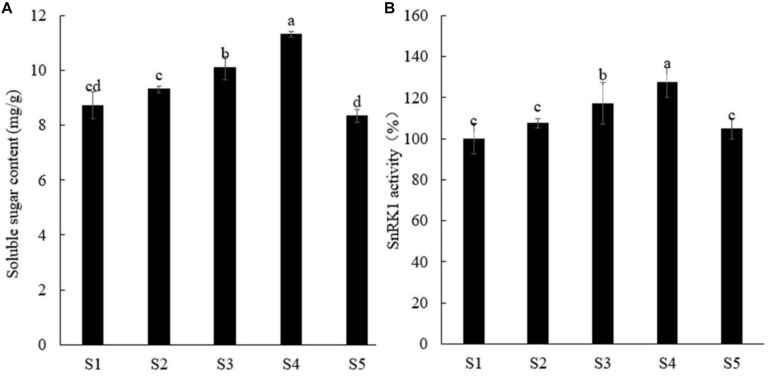
Effects of different concentrations of sucrose on soluble sugar content **(A)** and SnRK1 activity **(B)** in peach roots. Error bars represent the averages of three biological replicates ± SD. Different letters represent differences between different processes. Significance was defined at *P* < 0.05. S1, water; S2, 1% sucrose solution; S3, 3% sucrose solution; S4, 5% sucrose solution; S5, 7% sucrose solution.

Sugar not only is an important energy source for plants but also can be used as a signal molecule to participate in the process of plant growth and development (Tsai and Gazzarrini, 2014; Ljung et al., 2015). Liu et al. (2017) found that sucrose can promote the accumulation of anthocyanin and proanthocyanidin in apple, and *MdSnRK1.1* also plays an important role, indicating that SnRK1 plays a certain role in regulating plant growth in response to sugar signals. We first screened different sucrose concentrations and found that with increasing exogenous sucrose concentration, the soluble sugar content and SnRK1 enzyme activity in peach roots gradually increased, and 5% sucrose concentration had the best effect, whereas 7% sucrose showed inhibition (Figure 1), and the growth of peach roots showed the same trend (Supplementary Figure S1).

### Sucrose Promotes the Growth and Development of Peach Root System

**FIGURE 2:**
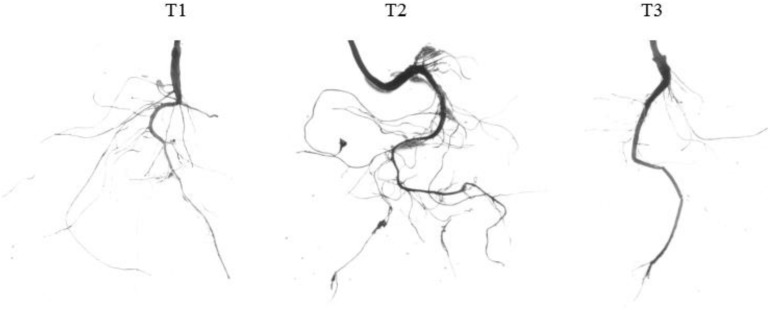
Effects of exogenous sucrose on the root phenotype of peach seedlings. T1, water; T2, 5% sucrose solution; T3, 5% mannitol solution.

Previous studies have found that exogenous sucrose can promote root growth of *A. thaliana* (Walton et al., 2007; Macgregor et al., 2008). Using water and mannitol as controls, we found that 5% sucrose enhanced root growth by significantly increasing the number of the first and second lateral roots, promoting root extension, increasing the total root length, and increasing the total surface area. Compared with water, the number of primary lateral roots, the number of secondary lateral roots, the total length of the root system, and the total surface area of the root system increased by 77.58, 173.31, 70.45, and 81.46%, respectively, after sucrose treatment. Mannitol could not induce root growth (Figure 2 and Table 2). These findings indicate that sucrose can regulate peach root growth, especially the development of lateral roots.

**TABLE 2:** Effects of exogenous sucrose on the root architecture of peach seedlings.

Experimental treatment	Number of primary lateral roots	Number of secondary lateral roots	Total root length (cm)	Total root surface area (cm^2^)
T1	235 ± 14.422^b^	123.67 ± 7.024^b^	86.627 ± 6.011^b^	9.8201 ± 1^b^
T2	417.33 ± 14.468^a^	338 ± 12.767^a^	147.66 ± 14.989^a^	17.82 ± 1.492^a^
T3	107.67 ± 8.963^c^	62.67 ± 7.638^c^	67.6684 ± 3.709^c^	6.75 ± 0.541^c^

*Error bars represent the averages of three biological replicates ± SD. Different letters represent differences between different processes. Significance was defined at *P* < 0.05.*

### Sucrose Increases the Auxin Content and Auxin-Related Gene Expression in Peach Root System

**FIGURE 3:**
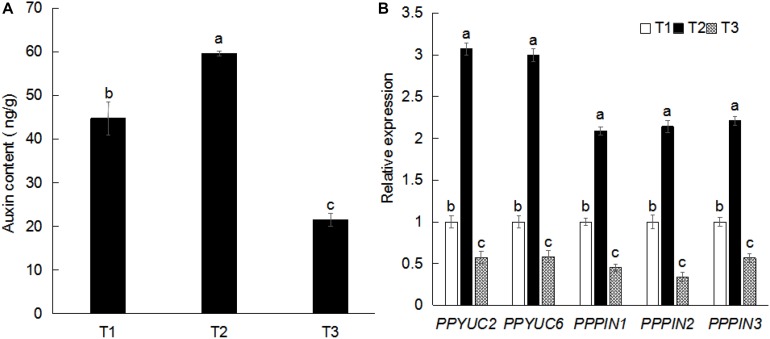
Effects of exogenous sucrose on auxin content **(A)** and gene expression **(B)** in plant roots. Error bars represent the averages of three biological replicates ± SD. Different letters represent differences between different processes. Significance was defined at *P* < 0.05. T1, water; T2, 5% sucrose solution; T3, 5% mannitol solution.

Auxin plays important roles in the growth and development of plant root system. To examine the effect of exogenous sucrose on root configuration, we measured the auxin content in peach root system. Compared with water, sucrose increased the IAA content by 34.04%. Compared with mannitol, sucrose increased the IAA content nearly twofold (Figure 3A). The expression of auxin-related genes in peach roots was examined by qPCR. As shown in Figure 3B, the expression of auxin synthesis-related genes (*PPYUC2*, *PPYUC6*) and auxin transport-related genes (*PPPIN1*, *PPPIN2*, and *PPPIN3*) (Petrasek, 2006; Kim et al., 2013) increased significantly after sucrose treatment, whereas their levels decreased after mannitol treatment. These findings reveal that the effect of exogenous sucrose on the root system may be caused by the up-regulation of auxin-related genes, thereby resulting in auxin accumulation in the root system.

### Peach SnRK1 Responds to Sucrose and Regulates Root Growth and Development

**FIGURE 4:**
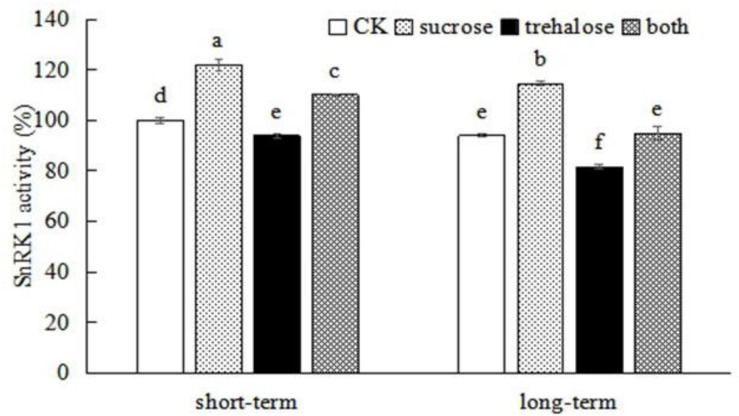
Exogenous sucrose affects SnRK1 enzyme activity in peach roots. Error bars represent the averages of three biological replicates ± SD. Different letters represent differences between different processes. Significance was defined at *P* < 0.05.

SnRK1, as an energy sensor, can respond to sucrose and regulate plant growth and development (Jossier et al., 2009; Liu et al., 2017). To examine whether peach SnRK1 is involved in sucrose-mediated root growth, SnRK1 activity was inhibited by trehalose. We found that short-term and long-term treatment with trehalose could reduce SnRK1 enzyme activity in roots (activity was reduced by 6.12% after short-term treatment and by 13.14% after long-term treatment), and sucrose could reverse the inhibitory effect of trehalose on SnRK1 enzyme activity (Figure 4). These findings indicate that sucrose affects peach SnRK1 and an appropriate concentration of sucrose can promote SnRK1 activity.

**FIGURE 5:**
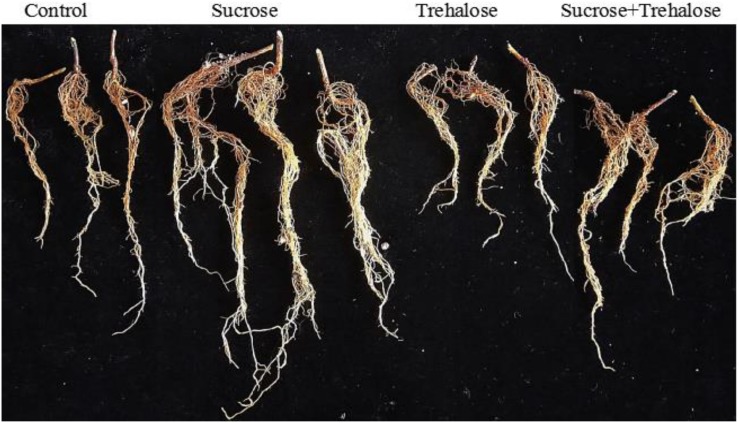
Effects of different treatments on the root phenotype of peach seedlings.

Based on the phenotype of the root system, trehalose could inhibit root growth, especially the number and growth of lateral roots. Compared with the control group, there was no significant difference in the total surface root area after trehalose treatment, but the total root length and the lateral root number were significantly decreased. Treatment with 5% sucrose could promote root growth and reverse the inhibitory effect of trehalose on root growth, in which the greatest effect was observed in lateral roots (Figure 5 and Table 3). These findings are consistent with those from SnRK1 enzyme activity experiments, indicating that sucrose promotes root growth because SnRK1 activity was altered in plant roots.

**TABLE 3:** Effects of different treatments on the root architecture of peach seedlings.

	Control	Sucrose	Trehalose	Sucrose + trehalose
The total root length (cm)	105.94 ± 3.61^b^	207.02 ± 11.46^a^	75.45 ± 12.02^c^	120.87 ± 4.40^b^
The root surface area (cm^2^)	10.03 ± 0.28^b^	13.89 ± 1.44^a^	8.60 ± 1.31^b^	10.34 ± 1.50^b^
Number of second link	535.33 ± 14.05^c^	1813.33 ± 94.50^a^	479.33 ± 13.87^c^	828 ± 48.00^b^
Number of third link	424 ± 10.44^c^	1333.67 ± 42.50^a^	334.33 ± 45.79^d^	803.67 ± 17.50^b^

*Error bars represent the averages of three biological replicates ± SD. Different letters represent differences between different processes. Significance was defined at *P* < 0.05.*

### Overexpression of *PpSnRK1*α Increases the Lateral Root Number and Changes the Root Configuration of *Arabidopsis thaliana*

**FIGURE 6:**
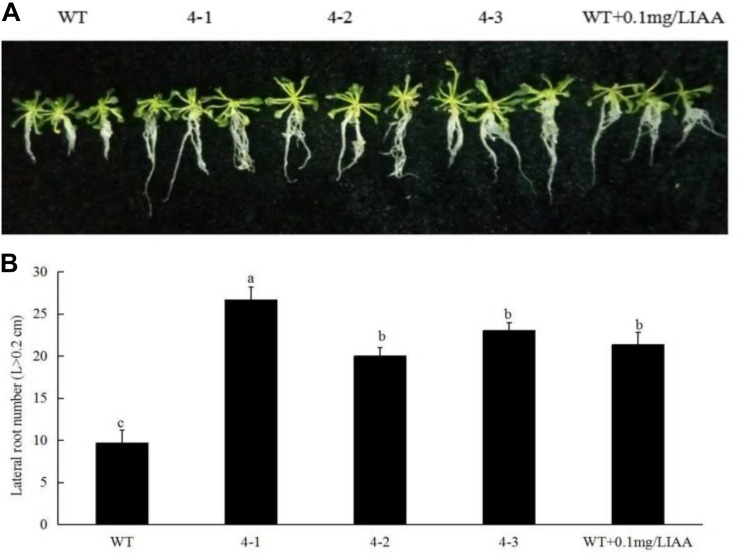
Effects of *PpSnRK1*α overexpression on the root architecture **(A)** and lateral root number **(B)** in *Arabidopsis thaliana.* Error bars represent the averages of three biological replicates ± SD. Different letters represent differences between different processes. Significance was defined at *P* < 0.05.

To prove that the change in SnRK1 enzyme activity could alter the root phenotype of plants, we produced peach *PpSnRK1*α-overexpressing homozygous lines. We found that root growth in *A. thaliana PpSnRK1*α-overexpressing plants (4-1, 4-2, and 4-3) was better than that of the WT, and the root length and surface area were both larger than those of the WT. Compared with the WT, the lateral root number increased 1.76 (4-1), 1.07 (4-2), and 1.38 (4-3) times in *PpSnRK1*α-overexpressing plants (Figure 6). These findings reveal that the number of lateral roots can be increased. This prompted us to investigate whether the effect of sucrose on the root configuration is caused by the increase in SnRK1 enzyme activity in plants. When WT plants were grown in MS medium supplemented with IAA, the lateral root number increased in the WT, indicating that auxin signaling is indeed involved in the growth and development of plant roots (Figure 6). These findings indicate that SnRK1 is involved in sucrose-mediated root growth and development through auxin signaling.

### Overexpression of *PpSnRK1*α Increases the Expression of Genes Involved in Auxin Synthesis and Auxin Transport in Roots

**FIGURE 7:**
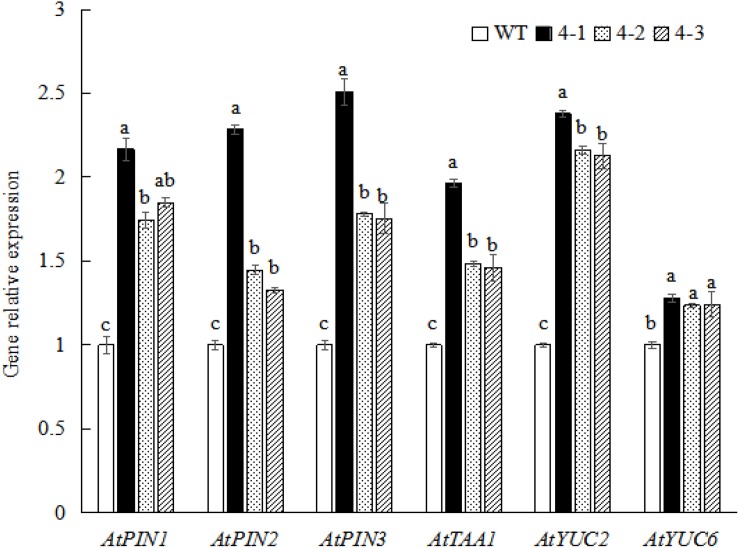
Auxin-related gene expression in *Arabidopsis thaliana* wild-type (WT) and *PpSnRK1*α-overexpressing (4-1, 4-2, and 4-3) plants. Error bars represent the averages of three biological replicates ± SD. Different letters represent differences between different lines in each gene. Significance was defined at *P* < 0.05.

To determine whether SnRK1 regulates auxin signaling and participates in sucrose-mediated root growth and development, the expression of auxin-related genes in *A. thaliana* WT and *PpSnRK1*α-overexpressing homozygous strains (4-1, 4-2, and 4-3) was examined. The results were consistent with those of the sucrose treatment experiment. The expression of auxin synthesis-related genes (*AtTAA1*, *AtYUC2*, and *AtYUC6*) and auxin transport-related genes (*AtPIN1*, *AtPIN2*, and *AtPIN3*) was significantly up-regulated compared with the WT, in which the expression of genes in the 4-1 homozygous strain was most significantly up-regulated (Figure 7). These findings reveal that SnRK1 is involved in auxin signaling and sucrose-mediated root growth and development.

### PpSnRK1 Interacts With IAA12 and PIN-LIKES6 in Yeast Two-Hybrid Experiments

**FIGURE 8:**
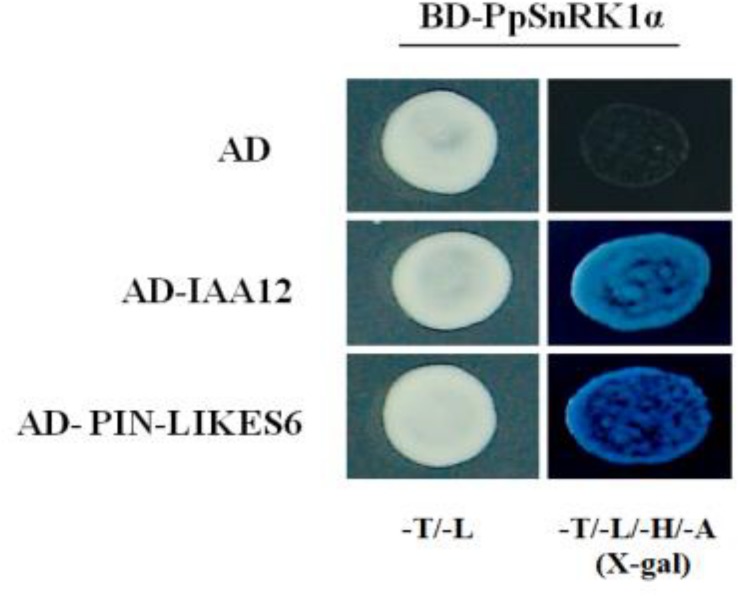
Results of the yeast two-hybrid assay showing the interactions between PpSnRK1α and IAA12/PIN6. Results are from one representative experiment out of three independent experiments.

Using peach PpSnRK1α as the bait, we screened the peach cDNA library and obtained two positive clones, IAA12 (ppa009545m) and PIN-LIKES6 (ppa006242m). Previous studies have reported that both proteins participate in auxin signaling and regulate root growth and development (Blanc, 2004; Blilou et al., 2005). The interactions between PpSnRK1α and IAA12/PIN-LIKES6 were determined by yeast two-hybrid assays. As shown in Figure 8, when BD-PpSnRK1α and AD-IAA12/AD-PIN6-LIKES6 were co-cultured in yeast, the colonies grew normally on the quadruple selection SD medium, and the spots turned blue, indicating that PpSnRK1α interacts with IAA12 and PIN6-LIKES6.

### PpSnRK1 Interacts With IAA12 and PIN-LIKES6 in the Bimolecular Fluorescence Complementation Assay

**FIGURE 9:**
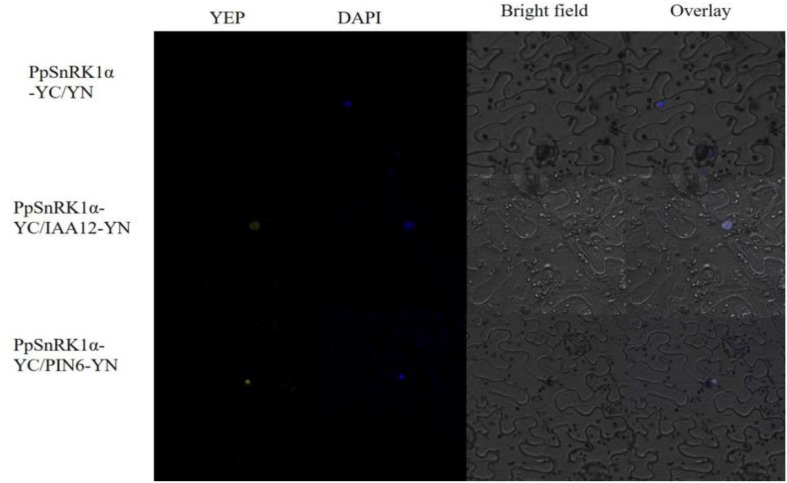
Results of the bimolecular fluorescence complementation (BiFC) assay showing the interaction between PpSnRK1α and IAA12/PIN6. The protein complex was visualized in tobacco leaf epidermal cells. Results are from one representative experiment out of three independent experiments.

The results of the bimolecular fluorescence complementation (BiFC) assay revealed that the incubation of PpSnRK1α-pSPYCE with IAA12-pSPYNE or PIN-LIKES6-pSPYNE in tobacco leaves for 2 days yielded a yellow fluorescent protein (YFP) signal, as observed in the nuclei of tobacco epidermal cells, and the YFP signal and blue fluorescent signal (nuclear location) were superimposable, indicating that PpSnRK1α interacts with IAA12 and PIN-LIKES6 in the nucleus (Figure 9).

### Sucrose Can Promote the Expression of Genes Encoding SnRK1 Interaction Protein *IAA12* and *PIN-LIKES6*

**FIGURE 10:**
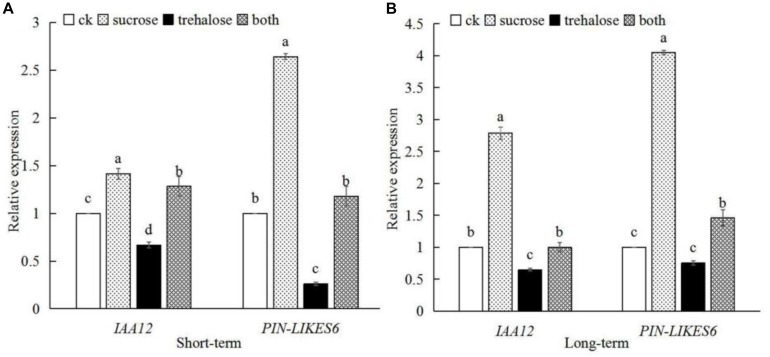
Effects of different treatments on the expression of *IAA1*2 and *PIN-LIKES6* genes in peach roots. **(A)** Gene expression after short-term treatment. **(B)** Gene expression after long-term treatment. Error bars represent the averages of three biological replicates ± SD. Different letters represent differences between different lines in each gene. Significance was defined as *P* < 0.05.

SnRK1 can respond to sucrose signals, interact with IAA12 and PIN-LIKES6, regulate auxin signal, and promote root growth and development. We further determine the *IAA12* and *PIN-LIKES6* gene expression (Figure 10), showing that the expression of these two genes was significantly up-regulated by sucrose in either short-term or long-term treatment, whereas the expression levels of the two genes decreased after trehalose inhibited SnRK1 enzyme activity. However, when sucrose and trehalose were applied together, sucrose could greatly relieve the inhibition effect of trehalose on gene expression. The expression trend of the two genes was consistent with SnRK1 dynamics after sucrose treatment (Figure 4). The above experiments strongly suggest that SnRK1 functions as an intermediate hub between sugar and auxin signaling to regulate the growth and development of plant roots.

## Discussion

Lateral roots can greatly increase the absorption area of roots; furthermore, the quantity, distribution, and development of roots directly affect the absorption of water and fertilizer (Ma et al., 2001; Fan and Yang, 2007). Plant hormones, such as auxin, as well as nutrients and the environment, can affect lateral root development (Fukaki and Tasaka, 2009; Malamy, 2005). Sugar, an important product of photosynthesis, is a substrate that not only provides energy for plant growth and development but also regulates related gene expression and enzyme activity as a signal molecule, thus regulating plant growth, development, and stress responses (Rolland et al., 2006; Li and Sheen, 2016). A previous study has reported that exogenous sugar plays an important role in lateral root formation in *A. thaliana* (Gupta et al., 2015). Sairanen et al. (2012) demonstrated that sucrose affects the transport and distribution of auxin and regulates the elongation of the hypocotyl, suggesting that sugar and auxin signaling are both involved in plant root development. However, there is no in-depth study on whether there is a synergistic effect between them on the regulation of root growth of fruit trees. In this study, 5% sucrose treatment was found to significantly increase the number of primary lateral roots, the number of secondary lateral roots, the total root length, and total surface area, which were significantly higher in treated seedlings than those in controls. Moreover, exogenous sucrose could up-regulate the expression of auxin synthesis-related and auxin transport-related genes in the root system and increase the auxin content. Taken collectively, these findings indicate that sugar and auxin signaling promote the growth and development of plant roots. It is important to note that our experiments show that 1, 3, and 5% sucrose concentration can promote the generation and growth of seedling root of peach, with 5% sucrose generating the best effect. However, after 7% sucrose treatment, the root growth of peach seedlings was inhibited, and the decreased trend of SnRK1 enzyme activity in the root of plants was consistent with that. Seven percent sucrose concentration inhibited the activity of SnRK1 enzyme (Figure 1 and Supplementary Figure S1).

The structure of SNF1/AMPK/SnRK1 protein kinase is highly conserved in all eukaryotes. It consists of a heterotrimer complex of α, β, and γ subunits (Hedbacker and Carlson, 2008; Carling et al., 2012; Crepin and Rolland, 2019), and it can regulate the metabolism of glucose and affect the growth and development of plants (Piattoni et al., 2011; Nukarinen et al., 2016; Wurzinger et al., 2018). SnRK1 activity is regulated by phosphorylation and dephosphorylation. Phosphorylation of the conserved threonine in the T-loop of its α catalytic subunit kinase domain activates SNF1/AMPK/SnRK1; that is, the *SnRK1*α subunit is critical for SnRK1 activity (Stein et al., 2000; McCartney and Schmidt, 2001; Baena-González et al., 2007; Shen et al., 2009; Crozet et al., 2010). In this experiment, it was found that the activity of SnRK1 enzyme was indeed up-regulated in *PpSnRK1*α-overexpressing plants (Supplementary Figure S2). It is well known that T6P can inhibit the activity of SnRK1 (Zhang et al., 2009; Debast et al., 2011). Schluepmann et al. (2003) found that when WT *Arabidopsis* was grown in excess exogenous trehalose, T6P accumulated in the plant. Delatte et al. (2011) also found that trehalose could increase T6P in plant roots, thereby inhibiting SnRK1 activity. We applied trehalose externally to indirectly inhibit SnRK1 enzyme activity, and we explored whether sucrose could restore its activity. It was found that trehalose could indeed inhibit the activity of SnRK1 enzyme in peach roots (Figures 4, 5 and Table 3) whereas sucrose treatment reversed the inhibitory effects of trehalose on SnRK1 enzyme activity and root growth. Although it was not clear whether it was only due to the intermediate effect of SnRK1, the positive regulation of root growth by SnRK1 could not be ruled out. In addition, when peach *PpSnRK1*α was overexpressed in *A. thaliana*, the total root surface area and the number of lateral roots were significantly increased, and the genes related to the synthesis and transport of auxin in the root of the overexpressed plants were also up-regulated, which was consistent with the trend of sucrose treatment. It indicates that peach SnRK1 can act as an intermediate messenger between sucrose and auxin signaling to regulate root growth and development.

Aberrant auxin synthesis and transport affect the root network as well as root growth (Boerjan et al., 1995; Reinhardt and Kuhlemeier, 2000; Swarup et al., 2005; Dubrovsky, 2008). Previous studies have reported that AUX/IAA, an early auxin response factor, plays key roles in root growth and development. For example, IAA14, IAA9, IAA1, IAA12, and other members are all involved in the growth and development of roots in different plants (Fukaki et al., 2002; Smet and Beeckman, 2010; Sha et al., 2015). Recent studies have found that *A. thaliana* mutant *iaa13* reduced lateral roots, which affected root configuration (Takaki et al., 2019). In addition, auxin output vectors composed of PIN family proteins, such as PIN1, PIN2, PIN3, PIN4, PIN5, PIN6, PIN7, and PIN8, are distributed in different tissues, where they regulate auxin synthesis and affect plant growth and development (Gälweiler et al., 1998; Friml and Palme, 2002; Blilou et al., 2005; Dubrovsky, 2008; Müller et al., 2014). In this study, we also screened two positive clones expressing IAA12 and PIN6 that were found to interact with PpSnRK1α. The proteins expressed in these clones possessed SnRK1 binding sites that were located in the nucleus, as demonstrated by the yeast two-hybrid assays and bimolecular fluorescence experiments. The expression levels of the two genes were determined by qRT-PCR technology, and it was found that sucrose significantly increase the expression of the two genes, whereas trehalose could significantly inhibit the expression of the two genes. When sucrose and trehalose were applied together, sucrose relieved the inhibition effect of trehalose. The results were consistent with the effect of sucrose on SnRK1 activity. This shows that SnRK1 as an intermediate signal connects sucrose and auxin signals and regulates the growth of peach root system.

**FIGURE 11:**
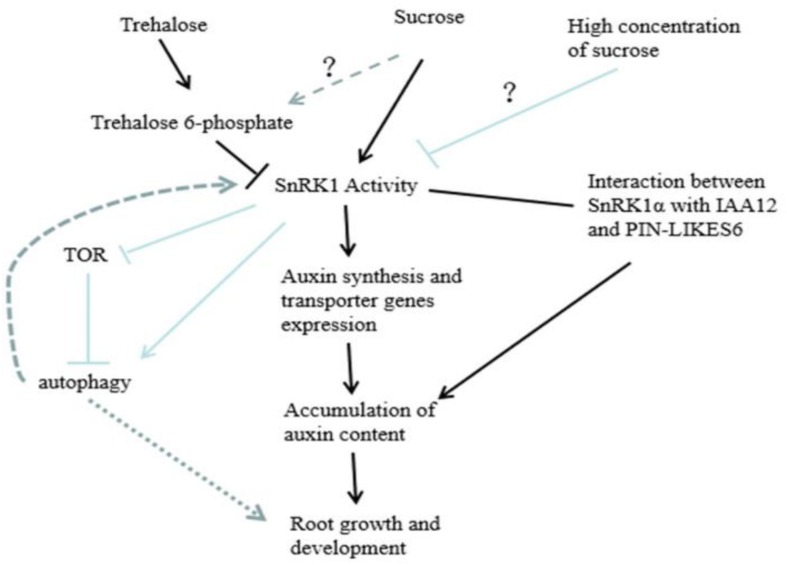
Proposed model showing how peach *PpSnRK1* participates in sucrose-mediated root growth through auxin signaling.

Through the analysis of the experimental data, it was found that the effect of sugar on root growth and development may be carried out through the pathway shown in Figure 11. SnRK1 can respond to sucrose, promote the expression of auxin synthesis-related and auxin transport-related genes, and interact with IAA12 and PIN-LIKES6 proteins in the auxin signaling pathway to regulate root growth and development. Sugar signaling is complex in plants, with concentration effects being highly variable between species and different organs (e.g., leaves vs. roots). In the case of peach roots, we observed that remarkably high exogenous sugar concentrations are required to inhibit SnRK1, as compared with other systems. Rodriguez et al. (2019) discussed the “yin and yang” model of TOR and SnRK1 signaling pathways in plants. That is, under feast conditions (sugar metabolism and energy balance), TOR is active while SnRK1 is inhibited. Under abiotic stress, SnRK1 is activated while TOR is inhibited, thus promoting catabolism and repressing anabolism (Robaglia et al., 2012; Broeckx et al., 2016; Baena-González and Hanson, 2017). However, the signaling of SnRK1 and TOR may depend on the autophagy and reactive oxygen species (ROS) pathways; that is, SnRK1 can activate autophagy, whereas TOR can inhibit autophagy. Moreover, autophagy also regulates the balance of sugar and ROS in plants (Janse van Rensburg et al., 2019; Signorelli et al., 2019). Whether it is regulated by the “yin and yang” model of TOR and SnRK1, the role of autophagy and ROS, as well as how the sugar of other components and hormone play a role in root growth and development, still needs to be further investigated. In addition, Fichtner et al. (2017) found that sucrose can act on the growth of axillary buds by regulating T6P, so whether there is a synergism between the two during root growth and development remains to be further explored. In conclusion, this study showed that SnRK1 activity is stimulated up to 5% exogenous sucrose treatment, urging the sugar signaling community to reconsider the definition of “sugar feast” in different plant species and organs. This study increased our understanding on the role of sugar mediated SnRK1 control in roots, and especially in lateral root development controlled by auxins, boosting further research into SnRK1 as a hub at the crossroad between sugar and auxin signaling pathways.

## Data Availability Statement

All datasets generated for this study are included in the article/Supplementary Material.

## Author Contributions

FP and SZ conceived and designed the experiments. SZ, WW, and XW performed the experiments. YX and SZ contributed the reagents, materials, and data analysis. SZ and FP wrote the manuscript.

## Conflict of Interest

The authors declare that the research was conducted in the absence of any commercial or financial relationships that could be construed as a potential conflict of interest.

## Supplementary Material

FIGURE S1 Effects of different concentrations of sucrose on the phenotype of peach roots. S1: Water; S2: 1% Sucrose solution; S3: 3% Sucrose solution; S4: 5% Sucrose solution; S5: 7% Sucrose solution.

FIGURE S2 Effect of *PpSnRK1*α-overexpressing (4-1, 4-2, 4-3) on the activity of SnRK1 enzyme. Error bars represent the averages of three biological replicates ± SD. Different letters represent differences between different processes. Significance was defined at *P* < 0.05.
