# Supplementary figures and images for: Peach PpSnRK1 Participates in Sucrose-Mediated Root Growth Through Auxin Signaling
Source: Front Plant Sci. 2020 Apr 24;11:409. doi: 10.3389/fpls.2020.00409 (PMC7193671; doi:10.3389/fpls.2020.00409)

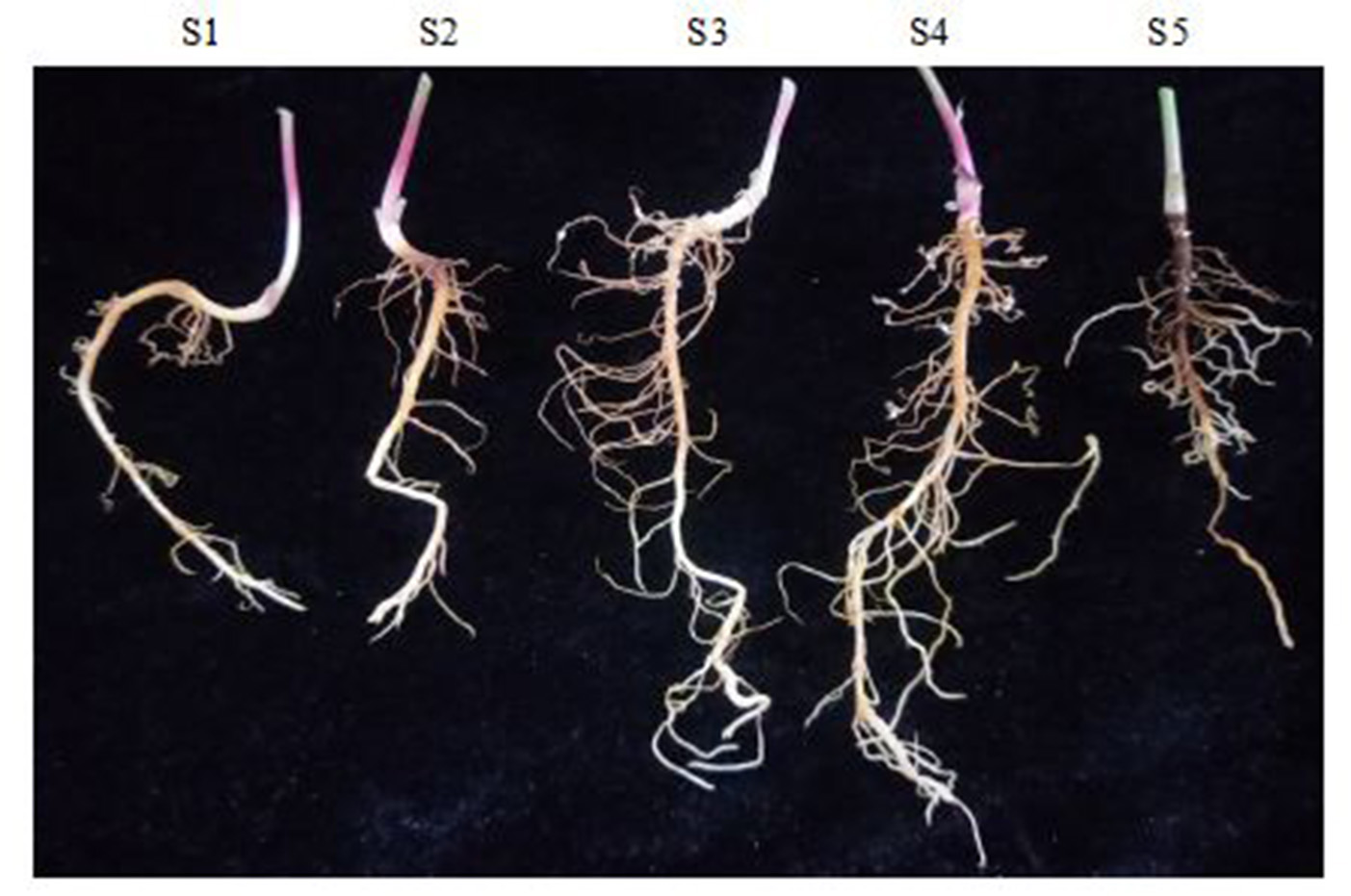

Supplement: FIGURE S1 — Effects of different concentrations of sucrose on the phenotype of peach roots. S1: Water; S2: 1% Sucrose solution; S3: 3% Sucrose solution; S4: 5% Sucrose solution; S5: 7% Sucrose solution. [file Image_1.jpg]

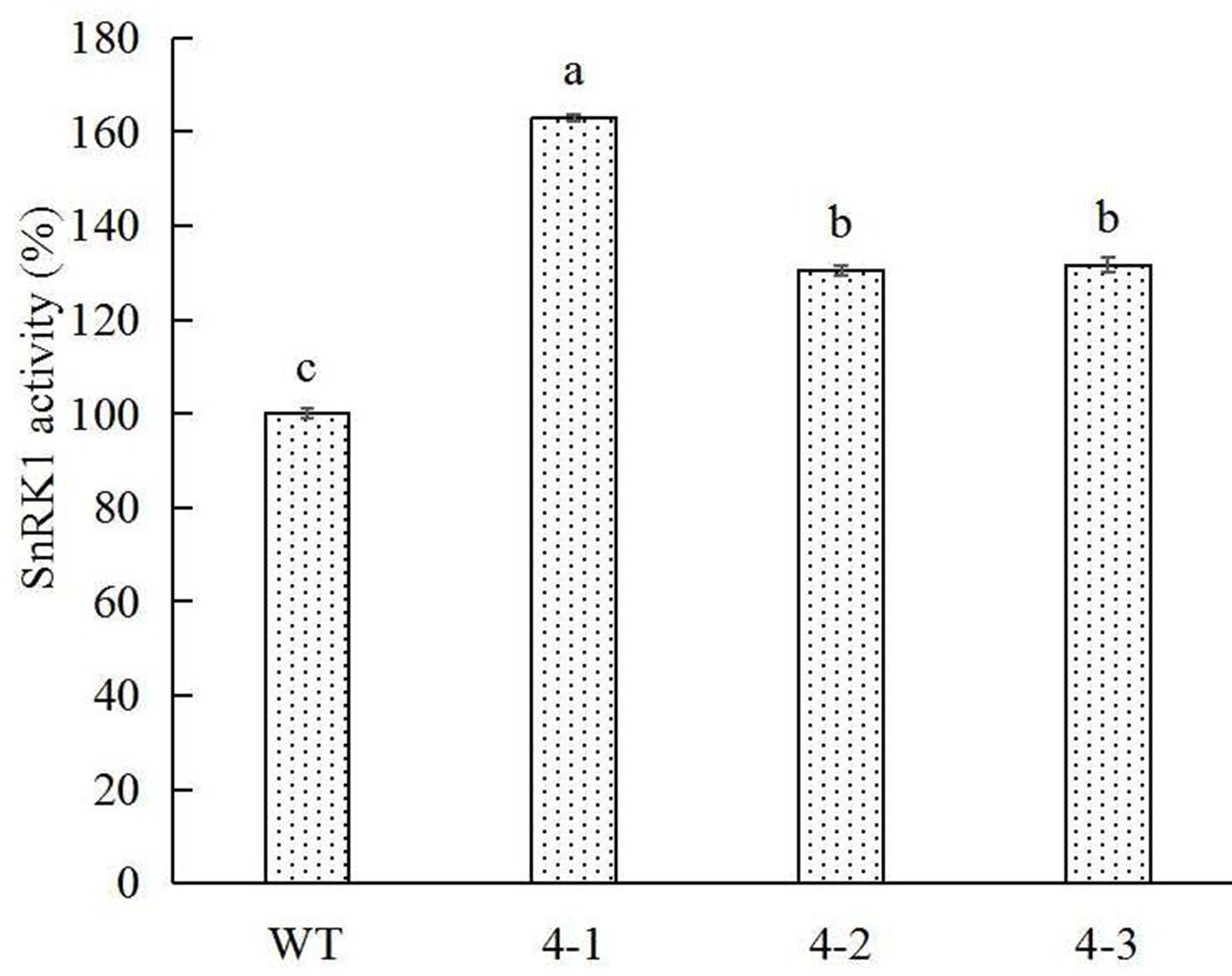

Supplement: FIGURE S2 — Effect of PpSnRK1α-overexpressing (4-1, 4-2, 4-3) on the activity of SnRK1 enzyme. Error bars represent the averages of three biological replicates ± SD. Different letters represent differences between different processes. Significance was defined at P < 0.05. [file Image_2.jpg]
